# The potential for improving cardio-renal outcomes by sodium-glucose co-transporter-2 inhibition in people with chronic kidney disease: a rationale for the EMPA-KIDNEY study

**DOI:** 10.1093/ckj/sfy090

**Published:** 2018-10-25

**Authors:** William G Herrington, David Preiss, Richard Haynes, Maximilian von Eynatten, Natalie Staplin, Sibylle J Hauske, Jyothis T George, Jennifer B Green, Martin J Landray, Colin Baigent, Christoph Wanner

**Affiliations:** 1 Medical Research Council Population Health Research Unit at the University of Oxford, Nuffield Department of Population Health, University of Oxford, Oxford, UK; 2 Clinical Trial Service Unit and Epidemiological Studies Unit, Nuffield Department of Population Health, University of Oxford, Oxford, UK; 3 Boehringer Ingelheim International, Ingelheim, Germany; 4 Li Ka Shing Centre for Health Information and Discovery, Big Data Institute, University of Oxford, Oxford, UK; 5 Duke Clinical Research Institute, Duke University Medical Center, Durham, NC, USA; 6 Würzburg University Clinic, Würzburg, Germany

**Keywords:** cardiovascular, CKD, clinical trial, diabetic kidney disease, SGLT-2 inhibitor

## Abstract

Diabetes is a common cause of chronic kidney disease (CKD), but in aggregate, non-diabetic diseases account for a higher proportion of cases of CKD than diabetes in many parts of the world. Inhibition of the renin–angiotensin system reduces the risk of kidney disease progression and treatments that lower blood pressure (BP) or low-density lipoprotein cholesterol reduce cardiovascular (CV) risk in this population. Nevertheless, despite such interventions, considerable risks for kidney and CV complications remain. Recently, large placebo-controlled outcome trials have shown that sodium-glucose co-transporter-2 (SGLT-2) inhibitors reduce the risk of CV disease (including CV death and hospitalization for heart failure) in people with type 2 diabetes who are at high risk of atherosclerotic disease, and these effects were largely independent of improvements in hyperglycaemia, BP and body weight. In the kidney, increased sodium delivery to the macula densa mediated by SGLT-2 inhibition has the potential to reduce intraglomerular pressure, which may explain why SGLT-2 inhibitors reduce albuminuria and appear to slow kidney function decline in people with diabetes. Importantly, in the trials completed to date, these benefits appeared to be maintained at lower levels of kidney function, despite attenuation of glycosuric effects, and did not appear to be dependent on ambient hyperglycaemia. There is therefore a rationale for studying the cardio-renal effects of SGLT-2 inhibition in people at risk of CV disease and hyperfiltration (i.e. those with substantially reduced nephron mass and/or albuminuria), irrespective of whether they have diabetes.

## INTRODUCTION

In high-income countries, the overall prevalence of chronic kidney disease (CKD) is ∼10% [1, 2] and this proportion is expected to rise as populations age further and diabetes mellitus becomes more common [3]. Worldwide, diabetic kidney disease accounts for a large proportion of advanced CKD (i.e. Stages 4 and 5), but the proportion of those without diabetes still ranges from ∼50 to 70% [4, 5]. CKD can often be a progressive condition, with proteinuria representing a significant risk factor for more rapid decline in kidney function [6]. The avoidance of progressive CKD is important, as end-stage kidney disease (ESKD) has adverse effects on morbidity and quality of life, dialysis or transplantation incur substantial societal costs [7, 8] and low levels of kidney function increase cardiovascular (CV) risk and premature mortality [9].

The current standard of care in many forms of CKD is inhibition of the renin–angiotensin system (RAS) with an angiotensin-converting enzyme inhibitor (ACEi) or angiotensin II receptor blocker (ARB). RAS inhibition has been shown to moderately reduce albuminuria and to slow the rate at which proteinuric kidney diseases progress [10–12]. Compared with RAS inhibition with a single drug, combination therapy (e.g. ACEi plus ARB) has not been shown to further delay kidney disease progression but does increase the risk of serious hyperkalaemia or acute kidney injury [13]. In people with diabetes, intensification of glycaemic control has been demonstrated to have moderately beneficial effects on markers of kidney disease progression compared with ‘standard’ regimens [14–16], and in the long-term this may translate into reduced risk of ESKD [17]. Trials of intensification of blood pressure (BP) lowering also suggest small benefits on CKD progression may perhaps exist among those with proteinuria [18–21]. Despite these interventions, however, substantial residual risk of ESKD remains.

Lowering low-density lipoprotein cholesterol has been shown to reduce the incidence of atherosclerotic events in people with CKD [22], and statin-based regimens are widely recommended for those at risk [23, 24]. A key feature of CKD, however, is the high prevalence of non-atherosclerotic heart disease. About half of patients with advanced CKD have abnormal cardiac structure [25, 26], increasing to more than three-quarters by the time dialysis is initiated [26, 27]. Although ejection fraction is often preserved, some degree of left ventricular diastolic dysfunction is common in CKD [25]. Lowering BP may reduce CV disease risk in CKD [28], but there is a general lack of reliable information about other treatments that may be effective for the prevention of heart disease in CKD [29]. Therefore, in addition to testing new interventions that could reduce the risk of CKD progression, there is a need for more trials of treatments that could further reduce the types of CV disease commonly experienced by people with CKD.

Sodium-glucose co-transporter-2 (SGLT-2) inhibitors were originally developed to treat hyperglycaemia in people with diabetes [30]. Recent large placebo-controlled outcome trials have shown that empagliflozin and canagliflozin reduce the risk of CV disease in people with type 2 diabetes mellitus (T2DM) at high risk of CV disease. Exploratory analyses also suggested they may reduce kidney disease progression in this population [31–33]. These CV and kidney effects appeared to be largely independent of effects on glycaemic control, BP and body weight.

In this review we introduce the mechanisms of SGLT-2 inhibition on the kidney and summarize the key clinical evidence providing a rationale for the testing the effects of SGLT-2 inhibition on kidney and CV outcomes in people with diabetic kidney disease. We then introduce the hypothesis that SGLT-2 inhibition may also have beneficial cardio-renal effects in people with CKD but without diabetes. Lastly, using randomized data from the Empagliflozin, Cardiovascular Outcomes, and Mortality in Type 2 Diabetes (EMPA-REG OUTCOME) trial, we describe what is currently known about the safety of empagliflozin in T2DM, both overall and in people with concomitant CKD.

## MECHANISMS OF ACTION AND PHYSIOLOGICAL EFFECTS OF SGLT-2 INHIBITORS ON THE KIDNEY

SGLT-2 is a kidney-specific solute transporter responsible for the vast majority (80–90%) of kidney tubular glucose reabsorption under normal physiological conditions [34]. Mutations that affect its function cause familial renal glycosuria, which appears to be a relatively benign condition [35]. In hyperglycaemic states, SGLT-2 expression can increase, causing more proximal tubular glucose and sodium reabsorption [36, 37]. The reduced sodium delivery to the distal convoluted tubules may result in a potentially maladaptive hyperfiltration state [30].

SGLT-2 inhibitors cause about half of filtered glucose to be excreted. This equates to ∼50–80 g/day under normoglycaemic and modest hyperglycaemic conditions and perhaps >100 g/day in people with diabetes and hyperfiltration [38]. Sodium-glucose co-transporter-1 (SGLT-1) is a distinct co-transporter that is present in the intestine, heart, skeletal muscle and kidney. It has a higher affinity but lower transporting capacity compared with SGLT-2. Nevertheless, increased SGLT-1 expression or activity in the distal segment of the proximal tubule in response to SGLT-2 inhibition may partly explain why a large proportion (perhaps 50%) of filtered glucose is still reabsorbed in those treated with an SGLT-2 inhibitor [39].

As each reabsorbed molecule of glucose by SGLT-2 is accompanied by a sodium ion [30], inhibition of SGLT-2 causes natriuresis in addition to the osmotic diuretic effect of glycosuria. This non-glycaemic effect may modify CV risk through reductions in plasma volume, organ congestion and central and systemic BP [40] and may be particularly beneficial at preventing heart failure (HF) [31, 32, 41, 42].

**FIGURE 1:**
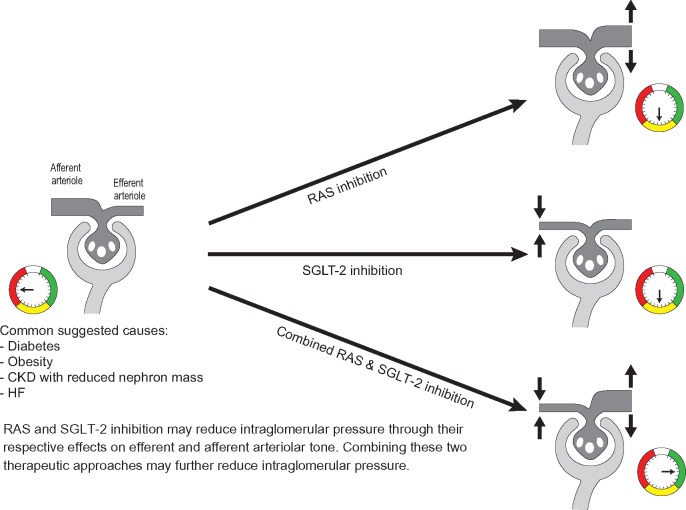
Mechanistic concept of the effects of RAS and SGLT-2 inhibition on intraglomerular pressure.

Natriuresis and altered tubular handling of filtered sodium may also have important modulatory effects on glomerular filtration rate (GFR) through reducing intraglomerular pressure [41, 43]. Hyperfiltration is driven in part by neurohormonal stimuli that cause either a net reduction in afferent glomerular arteriolar resistance or a net increase in efferent arteriolar resistance [43]. Angiotensin II is one key mediator of efferent arteriolar resistance and ACEi/ARBs effectively reduce intraglomerular pressure and slow kidney disease progression through a reduction of efferent vascular resistance [11, 12]. An alternative potential therapeutic strategy to reduce intraglomerular pressure might be to induce an increase in afferent arteriolar tone. Tubuloglomerular feedback and afferent arteriolar tone are attenuated in diabetes by up-regulation of tubular SGLTs and other sodium-exchange transport mechanisms. This leads to increased tubular sodium reabsorption and consequently to reduced delivery of sodium to the macula densa, where afferent arteriolar tone is relaxed in response to decreased adenosine production [41, 43]. SGLT-2 inhibition restores delivery of sodium to the macula densa, promoting adenosine production and increased afferent arteriolar tone (Figure 1). Some restoration of blunted tubuloglomerular feedback is apparent after a single dose, probably accounting for the acute reduction in GFR and the subsequent reduction in albuminuria [44] observed in people treated with SGLT-2 inhibitors, as well as the swift return towards pre-treatment levels of GFR shortly after drug discontinuation [33, 44]. Importantly, these effects do not appear to be modified by concomitant use of a RAS blocker [31, 33] or loop diuretic [45]. The latter observation may be of interest as loop diuretics block Na-K-Cl co-transporter (NKCC2) channels, which are the main mechanism by which chloride ions enter the macula densa to initiate tubuloglomerular feedback [46]. It is therefore hypothesized that additional mechanisms beyond NKCC2 sensing may also be mediating the renal haemodynamic effects of SGLT-2 inhibition.

Attenuation of tubuloglomerular feedback and intraglomerular ‘hypertension’ are not unique to diabetes. Activation of the sympathetic nervous system has been shown to increase proximal tubular sodium reabsorption in HF [47] and is recognized in people with pre-diabetes, pre-hypertension [48] and obesity [49]. Moreover, among those with reduced nephron mass, which includes many people with chronically reduced GFR, intrarenal vasodilatation may explain why the remaining nephrons undergo structural hypertrophy and single-nephron GFR increases [43, 50].

Intraglomerular hypertension has long been considered to be a final common pathway for kidney disease progression shared by many forms of CKD [51]. Support for this hypothesis includes the observations that for a given level of urinary albumin excretion, the risk of ESKD is relatively independent of the primary cause of kidney disease [52] and RAS blockade appears effective at slowing both the progression of non-diabetic [10, 53] and diabetic [11, 12] proteinuric kidney diseases. SGLT-2 inhibition may restore tubuloglomerular feedback and reduce intraglomerular pressure through non-glycaemic mechanisms in people with diabetes (including those already on RAS blockade) [31, 33, 44] and has acute effects on GFR in people without diabetes [54–56]. It is therefore reasonable to hypothesize that in people who are at high risk of hyperfiltration (i.e. those with albuminuria and/or low GFR), SGLT-2 inhibition may lower intraglomerular pressure and be nephroprotective even in the presence of ambient normoglycaemia.

## RANDOMIZED TRIAL EVIDENCE FOR EFFECTS OF SGLT-2 INHIBITION ON INTERMEDIATE FACTORS

### Effects of SGLT-2 inhibition on glycaemic control and urinary glucose excretion

In people with T2DM, trials have established the glucose-lowering effects of SGLT-2 inhibition, and there appears to be little difference between empagliflozin, canagliflozin and dapagliflozin in this regard [57]. In short- and medium-term mechanistic trials, on average, adding SGLT-2 inhibition to various background glucose-lowering therapies reduced glycosylated haemoglobin (HbA1c) by ∼0.7% [58], with absolute reductions in HbA1c often larger among those with the highest baseline HbA1c [59, 60].

In people with T2DM and CKD, previous pharmacodynamic studies have consistently shown a linear relationship between 24-h urinary glucose excretion and kidney function [41]. Consequently, compared with those with preserved GFR, HbA1c reductions with SGLT-2 inhibition are smaller in people with lower GFR. In pooled analyses of randomized trials comparing dapagliflozin or empagliflozin versus placebo among people with T2DM and estimated GFR (eGFR) levels <60 mL/min/1.73 m^2^, HbA1c differences were generally attenuated to −0.3 and −0.4%, respectively [61, 62].

In people without diabetes, studies have found that empagliflozin 25 mg/day and canagliflozin 300 mg/day each induce ∼50–60 g/day of urinary glucose excretion [55, 63], which is approximately half the corresponding amount induced in people with diabetes [64].

### Effects of SGLT-2 inhibition on body fat and whole-body fluid homeostasis

Glycosuria induced by SGLT-2 inhibition provides a direct source of calorie loss, but there are also indirect metabolic responses induced that are expected to enhance lipolysis. These include a decrease in insulin and an increase in glucagon secretion, resulting perhaps from both the expected reduction in plasma glucose and effects on both pancreatic alpha and beta cells [65]. The metabolic consequence is a shift to more utilization of fat for energy production. Modestly increased plasma glycerol and fatty acid levels (reflecting accelerated lipolysis) and higher levels of the ketone β-hydroxybutyrate (reflecting higher liver fat oxidation) are apparent within 2 weeks of starting empagliflozin, and these effects, although perhaps attenuated, are also present in people without diabetes [54].

Together, these metabolic effects lead to reductions in body weight [66], including loss of visceral and subcutaneous adipose tissue [67]. Although there are effects on fluid homeostasis, after 2 years, weight loss resulting from SGLT-2 inhibition in T2DM appears attributable in large part to reduced adipose tissue (measured using dual-energy X-ray absorptiometry) [67]. In the EMPA-REG OUTCOME trial, empagliflozin 10–25 mg/day led to a sustained difference in weight of about −2 kg (from a mean of 86 kg) and a −2 cm difference in waist circumference compared with placebo (from a mean of 105 cm) in 7020 people with T2DM and prior atherosclerotic CV disease [32]. There were almost identical-sized reductions observed with canagliflozin 100–300 mg versus placebo among the 10 142 participants in the Canagliflozin Cardiovascular Assessment Study (CANVAS) and CANVAS-Renal (CANVAS-R) [31]. However, fat loss is less than predicted from estimated urinary calorie loss, perhaps because participants allocated active drug were more likely to increase calorie intake than those allocated placebo [68].

Nevertheless, despite the reduced glycosuric effects of SGLT-2 inhibition in people with T2DM and CKD, lower eGFR has not been shown to attenuate these weight-lowering effects, at least within the range of eGFR studied to date. In placebo-controlled trials testing dapagliflozin [61, 69] and empagliflozin [62] in T2DM, differences in weight among those with preserved kidney function were similar to differences observed in those with CKD Stages 3A, 3B or 4. Part of the preserved effect of SGLT-2 inhibition on body weight in CKD may therefore result from increased urinary sodium and electrolyte free-water excretion [70] (although non-renal effects centrally or in the gut cannot be excluded).

In a mathematical extrapolation of a short-term randomized trial in healthy individuals, dapagliflozin was shown to cause proportionally larger reductions in interstitial fluid volume (−480 mL) than blood volume (−150 mL), which is in contrast to the effects of the loop diuretic bumetanide, which caused smaller reductions in interstitial fluid volume (−510 mL) than blood volume (−780 mL) [70]. Relatively larger reductions in interstitial fluid compared with plasma volume, perhaps the result of combined natriuretic and osmotic diuretic effects of SGLT-2 inhibition, may have the potential to significantly improve organ congestion in people with fluid overload with less risk of causing arterial underperfusion or symptomatic dehydration [70], but studies directly measuring changes in interstitial and blood volume in people with CKD and people with HF are needed to test both the mathematical extrapolation and these hypotheses.

### Effects of SGLT-2 inhibition on BP

Short- and medium-term mechanistic trials in T2DM show that SGLT-2 inhibitors produce modest reductions in office-measured and 24-h ambulatory BP [66, 71], central systolic BP and central pulse pressure [40]. A systolic BP difference of a similar size (−4 mmHg) to that measured in these smaller studies was observed in those allocated empagliflozin versus placebo in the EMPA-REG OUTCOME trial [32].

As is the case for body weight, the effects of SGLT-2 inhibition on BP are not diminished at lower eGFR levels, at least within the range of eGFR studied to date. For example, in 2286 people with T2DM randomized to empagliflozin versus placebo, BP reductions were at least as large even at an eGFR <30 mL/min/1.73 m^2^. Similarly, CKD Stages 3A and 3B have not been shown to modify the BP-lowering effect of dapagliflozin [61, 69]. The persistence of the BP-lowering effects of SGLT-2 inhibition in CKD is yet to be fully understood, and possibilities include BP being more responsive to salt mobilization and/or removal in CKD, increased response to the possible beneficial effects of SGLT-2 inhibition on arterial stiffness, sympathetic system overactivity, oxidative stress and endothelial dysfunction [62] and/or augmentation of the effect of other antihypertensive medications [62].

In people without diabetes, a randomized study of 376 obese individuals found that 12 weeks of canagliflozin 100–300 mg/day caused a systolic BP difference of between −1 and −2 mmHg compared with placebo [56]. Few randomized data are currently available in people with CKD but without diabetes, but the data from studies in other populations suggest that a modest BP-lowering effect of SGLT-2 inhibition might be expected in such people.

### Effects of SGLT-2 inhibition on albuminuria and short-term effects on GFR

In the EMPA-REG OUTCOME trial and CANVAS/CANVAS-R, both empagliflozin 10–25 mg/day and canagliflozin 100–300 mg/day were found to reduce albuminuria by between 25 and 50% in those with T2DM and either micro- or macroalbuminuria, irrespective of use of RAS blockade. The albuminuria-lowering effect was apparent early and maintained throughout the 3–4 years of follow-up [44, 72]. Furthermore, in the EMPA-REG OUTCOME trial, compared with those allocated to placebo, albuminuria remained lower among those allocated to empagliflozin about 1 month after stopping study treatment, suggesting that SGLT-2 inhibition prevented diabetes-related structural changes in the kidney (although this hypothesis has not been tested by kidney biopsy studies) [44].

**FIGURE 2:**
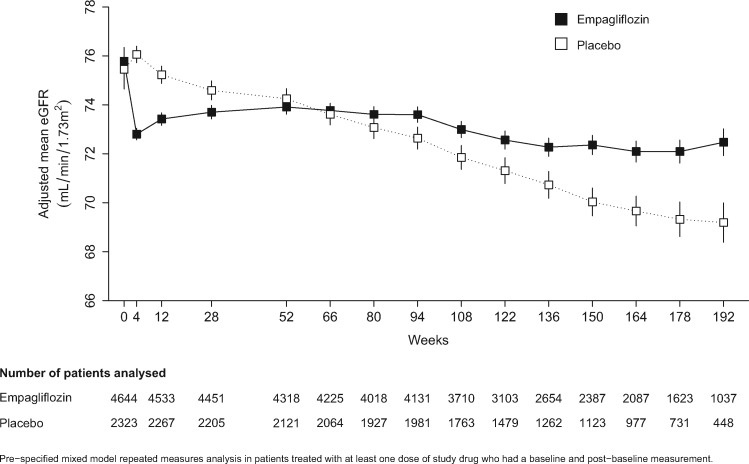
Effect of allocation to empagliflozin versus placebo on Chronic Kidney Disease Epidemiology Collaboration eGFR.

The EMPA-REG OUTCOME trial included people with T2DM with both preserved and modestly reduced kidney function. Commencement of SGLT-2 inhibition led to an initial acute decline in eGFR of ∼3 mL/min/1.73 m^2^. The magnitude of this effect was consistent at daily empagliflozin doses of 10 and 25 mg [33]. The initial dip in eGFR, which is considered an indicator of reduced intraglomerular pressure, was followed by a marked slower decline in eGFR compared with those allocated placebo during longer-term treatment (Figure 2) [33]. These findings have since been replicated in the CANVAS/CANVAS-R data [73].

Canagliflozin 100–300 mg/day has been compared with the sulfonylurea glimepiride in 1450 people with T2DM [72]. This comparison was particularly informative as the reductions in HbA1c were very similar among those allocated the two different classes of glucose-lowering drug. However, after an initial eGFR dip of ∼6 mL/min/1.73 m^2^, the average annual rate of decline in eGFR was 0.5–1.0 mL/min/1.73 m^2^/year among those allocated to canagliflozin compared with 3.3 mL/min/1.73 m^2^/year among those allocated to glimepiride (in whom no acute change in eGFR was observed). Such data further support the hypothesis that non-glycaemic effects of SGLT-2 inhibition are central to any nephroprotective effects.

In a subgroup analysis of the EMPA-REG OUTCOME trial, participants with reduced eGFR had a similar-sized initial eGFR dip followed by a relative slowing in the annual rate of decline in eGFR [33]. Similarly, data from five medium-term (i.e. 6 month) placebo-controlled trials of empagliflozin also confirm the initial dip in eGFR is at least as large in those with an eGFR between 30 and 60 mL/min/1.73 m^2^ compared with those with preserved kidney function. To date, too few people with an eGFR <30 mL/min/1.73 m^2^ have been studied to be certain, but there is some evidence that the initial eGFR dip is also present at this low level of kidney function [62].

In obese but otherwise healthy adults with normal kidney function, canagliflozin has been shown to cause an initial dip in eGFR, which in this population was ∼1–2 mL/min/1.73 m^2^ [56]. Two non-randomized studies in people without diabetes and with normal kidney function starting empagliflozin have also noted initial dips in eGFR of −8 to −16 mL/min/1.73 m^2^, respectively, although the lack of a control arm may mean these reflect substantial overestimates of effect [54, 55]. Nonetheless, this evidence suggests that lowering of intraglomerular pressure can be achieved without the need for ambient hyperglycaemia. There are no long-term published studies in non-diabetic populations exploring whether this initial dip in kidney function with SGLT-2 inhibition is associated with beneficial effects on subsequent eGFR slopes.

Taken together, these data on intermediate clinical parameters show that although the effects on HbA1c are attenuated among people with T2DM and lower kidney function, important non-glycaemic effects on fluid balance, body weight, BP, albuminuria and markers of intraglomerular pressure are present in individuals with reduced kidney function, at least down to an eGFR of 30 mL/min/1.73 m^2^. Although the effects of SGLT-2 inhibition on such parameters among those without diabetes remains less well studied to date, there is reason to expect pharmacological effects that may translate into clinical benefits.

## RANDOMIZED TRIAL EVIDENCE FOR EFFECTS OF SGLT-2 INHIBITION ON CLINICAL EFFICACY OUTCOMES

### Effects of SGLT-2 inhibition on kidney disease progression

In exploratory analyses from two large outcome trials, a hypothesis that SGLT-2 inhibition with empagliflozin or canagliflozin has the potential to reduce the risk of kidney disease progression in people with T2DM was raised. In the EMPA-REG OUTCOME trial, empagliflozin 10–25 mg/day reduced the incidence of the traditional renal composite outcome of doubling of creatinine (with eGFR ≤45 mL/min/1.73 m^2^), initiation of renal replacement therapy (RRT) or renal death by 46% {hazard ratio [HR] 0.54 [95% confidence interval (CI) 0.40–0.75]} [33]. This included a nominally significant reduction in the necessity to start RRT [HR 0.45 (95% CI 0.21–0.97)]. In the CANVAS/CANVAS-R, compared with placebo, canagliflozin 100–300 mg/day reduced the risk of the composite renal outcome of a 40% decline in eGFR, initiation of RRT or renal death by 40% [HR 0.60 (95% CI 0.47–0.77)] [31].

**FIGURE 3:**
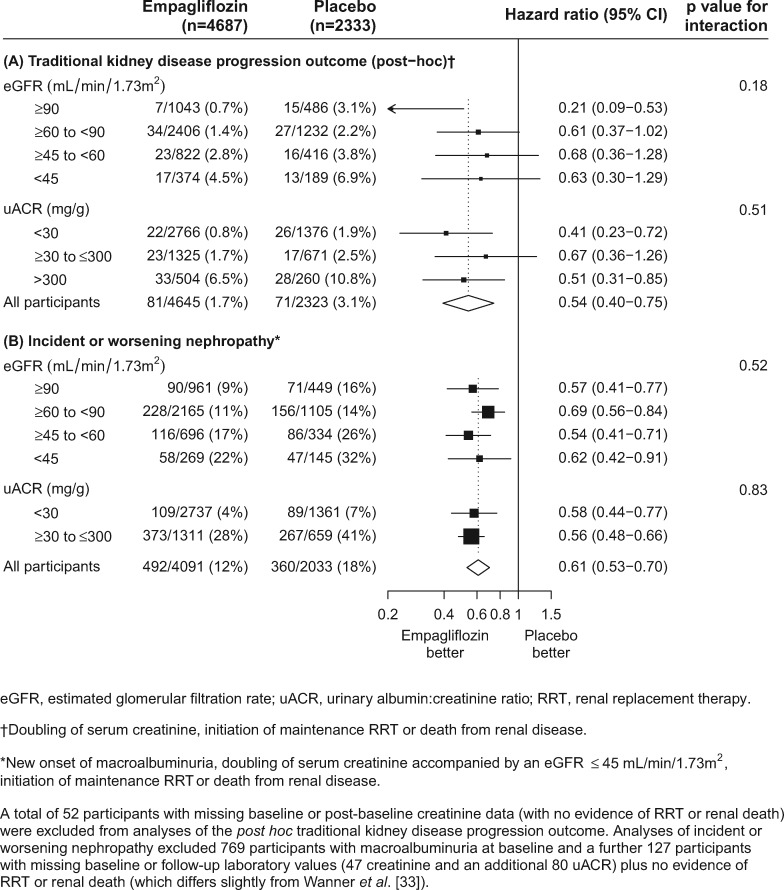
Effect of allocation to empagliflozin versus placebo on (**A**) traditional kidney disease progression outcome (*post hoc*) and (**B**) incident or worsening nephropathy, by baseline eGFR and uACR.

The EMPA-REG OUTCOME trial enrolled ∼1800 people with an eGFR <60 mL/min/1.73 m^2^, including ∼560 individuals with an eGFR <45 mL/min/1.73 m^2^. The proportional effects of empagliflozin on the traditional renal composite were similar irrespective of eGFR at baseline (interaction P = 0.18) or baseline levels of albuminuria (interaction P = 0.51; Figure 3A) [33]. These findings are reinforced by similar observations on a pre-specified renal composite outcome that further included new-onset macroalbuminuria (Figure 3B).

The first large-scale data on the effects of SGLT-2 inhibition on diabetic kidney disease progression will be provided by the Evaluation of the Effects of Canagliflozin on Renal and Cardiovascular Outcomes in Participants with Diabetic Nephropathy (CREDENCE) [74], which has recently been stopped early for benefit at the formal interim analysis planned for once 405 participants had experienced a primary outcome [75, 76]. CREDENCE includes 4401 people with T2DM and macroalbuminuria [urinary albumin:creatinine ratio (uACR) 300–5000 mg/g) and an eGFR of 30–90 mL/min/1.73 m^2^ on stable RAS blockade. The baseline mean eGFR was 56 mL/min/1.73 m^2^ and the median uACR was 927 mg/g. The treatment comparison is of canagliflozin 100 mg versus matching placebo [75], and the primary composite outcome is a doubling of serum creatinine, ESKD or death from renal or CV causes. Large-scale trials testing empagliflozin versus placebo (EMPA-KIDNEY) and dapagliflozin versus placebo (Dapa-CKD) in people with CKD are in progress [77]. Their designs differ from CREDENCE in that both these studies are looking at a CKD population with and without diabetes (Table 1), with EMPA-KIDNEY uniquely including people with a low eGFR (<45 mL/min/1.73 m^2^) with or without albuminuria.

**Table 1.** Ongoing large SGLT-2 inhibitor clinical trials in CKD and HF populations

	Key inclusion criteria	Size	Interventions	Primary outcomes	Selected secondary outcomes
CKD populations					
EMPA-KIDNEY: The Study of Heart and Kidney Protection with Empagliflozin [82]	Age ≥18 years eGFR 20–45 or eGFR 45–90 mL/min/1.73 m^2^ with uACR ≥200 mg/g Clinically appropriate doses of RAS blockade, unless not tolerated	5000 (≥1/3 with DM and ≥1/3 without DM)	Empagliflozin 10 mg/day versus placebo	Sustained ≥40% decline in eGFR, ESKD or death from renal or CV causes	CV death or hospitalization for HF All-cause hospitalization All-cause mortality
CREDENCE [74, 75]	Age ≥30 years T2DM, HbA1c 6.5–12% eGFR 30–90 mL/min/1.73 m^2^ Stable maximally tolerated RAS blockade uACR 300–5000 mg/g	4401	Canagliflozin 100 mg/day versus placebo	Doubling of creatinine, ESKD or death from renal or CV causes	CV death or hospitalization for HF Doubling of creatinine, ESKD or death from a renal cause
Dapa-CKD [77]	Age ≥18 years eGFR 25–75 mL/min/1.73 m^2^ Stable maximally tolerated RAS blockade, if not contraindicated uACR 200–5000 mg/g	4000	Dapagliflozin 5 or 10 mg/day versus placebo	Sustained ≥50% decline in eGFR, ESKD or death from renal or CV causes	CV death or hospitalization for HF Sustained ≥50% decline in eGFR, ESKD or death from a renal cause
HF populations					
EMPEROR-Preserved [88]	Age ≥18 years Symptomatic chronic HF with LVEF >40% NT-proBNP >300 pg/mL (or >900 if in AF) Stable dose of oral diuretic	4100	Empagliflozin 10 mg/day versus placebo	CV death or hospitalization for HF	eGFR slope Sustained ≥40% decline in eGFR or ESKD
EMPEROR-Reduced [89]	Age ≥18 years Class II–IV chronic HF with LVEF ≤40% NT-proBNP above a certain threshold (stratified by LVEF) Appropriate doses of medical therapy and use of medical devices	2800	Empagliflozin 10 mg/day versus placebo	CV death or hospitalization for HF	eGFR slope Sustained ≥40% decline in eGFR or ESKD
Dapa-HF [90]	Age ≥18 years Symptomatic chronic HF with LVEF ≤40% NT-proBNP ≥600 pg/mL eGFR ≥30 mL/min/1.73 m^2^ Appropriate background standard of care	4500	Dapagliflozin 10 mg/day versus placebo	CV death, hospitalization for HF or urgent HF visit	Sustained ≥50% decline in eGFR, ESKD or death from a renal cause

AF, atrial fibrillation; LVEF, left ventricular ejection fraction; NT-proBNP, N-terminal of the prohormone of brain natriuretic peptide.

Other large placebo-controlled SGLT-2 trials in those with T2DM and high CV risk include DECLARE (dapagliflozin 5 or 10 mg) [91], VERTIS CV (ertugliflozin 5 or 15 mg) [92] and SCORED [93] (sotagliflozin in those with an eGFR of 25–60 mL/min/1.73 m^2^) which are enrolling 17 276, ∼8000 and ∼10 500 people, respectively, and include kidney disease progression endpoints as secondary outcomes. DELIVER (Dapagliflozin 10 mg) in ∼4700 with preserved LVEF heart failure and SOLOIST-WHF (sotagliflozin) in ∼4000 people with heart failure and diabetes are also in development. CREDENCE has been stopped early for efficacy [76] and is likely to report in 2019. Both Dapa-CKD and EMPA-KIDNEY are event-driven trials and are expected to complete follow-up in around November 2020 and June 2022, respectively.

### Effects of SGLT-2 inhibition on CV diseases

The primary endpoint in the EMPA-REG OUTCOME trial was a CV composite of death from CV causes, non-fatal myocardial infarction or non-fatal stroke. Empagliflozin 10–25 mg was shown to reduce this composite by 14% compared with placebo [HR 0.86 (95% CI 0.74–0.99)] [32]. This effect was driven by a highly significant 38% [HR 0.62 (95% CI 0.49–0.77)] reduction in CV death. A beneficial effect of SGLT-2 inhibition on CV risk has also been observed in the CANVAS/CANVAS-R. Canagliflozin 100–300 mg/day reduced the primary composite outcome of death from CV causes, non-fatal myocardial infarction or non-fatal stroke by 14% [HR 0.86 (95% CI 0.75–0.97)] [31]. The effect on CV death in the CANVAS/CANVAS-R [HR 0.87 (95% CI 0.72–1.06)] was directionally consistent, although more modest, than in the EMPA-REG OUTCOME trial.

Given the effects of SGLT-2 inhibition on interstitial/plasma fluid volume [70] and BP [66, 71], a beneficial effect on cardiac pre-load and after-load is expected, and a reduction in the incidence of clinical outcomes among those with HF with reduced or preserved ejection fraction might be anticipated with such treatments [42]. Hormonal and metabolic effects of SGLT-2 inhibition may also mediate cardiac benefits. Intriguingly, increased ketone production may have beneficial effects on hypertrophied or failing hearts [78, 79].

In the EMPA-REG OUTCOME trial, the pre-specified secondary outcome of hospitalization for HF was reduced by 35% (HR 0.65 (95% CI 0.50–0.85)] [32] while allocation to canagliflozin in the CANVAS/CANVAS-R also reduced the risk of hospitalization for HF by about one-third compared with placebo [HR 0.67 (95% CI 0.52–0.87)] [31]. As HF with preserved ejection fraction is common in people with diabetes (and may even be more common in this population than HF with reduced ejection fraction [80]), this effect may have resulted in some reduction in the risk of HF in people with preserved ejection fraction [25, 81]. This is an important suggestion, as preserved ejection fraction HF, which is common in CKD, has few proven effective therapies [29]. However, neither of these trials definitively differentiated the type of HF, so it is not possible to confirm a reduced risk of preserved ejection fraction HF. Instead, the Empagliflozin Outcome Trial in Patients with Chronic Heart Failure with Preserved Ejection Fraction (EMPEROR-Preserved) and EMPEROR-Reduced trials with empagliflozin 10 mg versus placebo are recruiting from these two HF populations and including people with and without diabetes. In addition, the effects of dapagliflozin versus placebo in people with reduced ejection fraction are being explored in Dapa-HF (Table 1). The primary endpoints of all three of these dedicated HF trials include a composite of CV death or hospitalization for HF [42]. The three large kidney trials of SGLT-2 inhibition have included this same composite as a secondary outcome [74, 77, 82, 83]. It should be noted that in *post hoc* analyses of the EMPA-REG OUTCOME trial, allocation to empagliflozin reduced the risk of CV death or hospitalization for HF by 34% [HR 0.66 (95% CI 0.55–0.79)], a benefit that was similar irrespective of baseline risk of HF [84].

Exploration of the EMPA-REG OUTCOME data has suggested that the increase in haematocrit caused by empagliflozin, a surrogate for reductions in plasma volume, was the intermediate clinical parameter with the largest mediating effect on the reduction in CV death [85]. This observation may have particular relevance in CKD populations, where non-atherosclerotic heart disease and fluid overload/HF are common [80].

**FIGURE 4:**
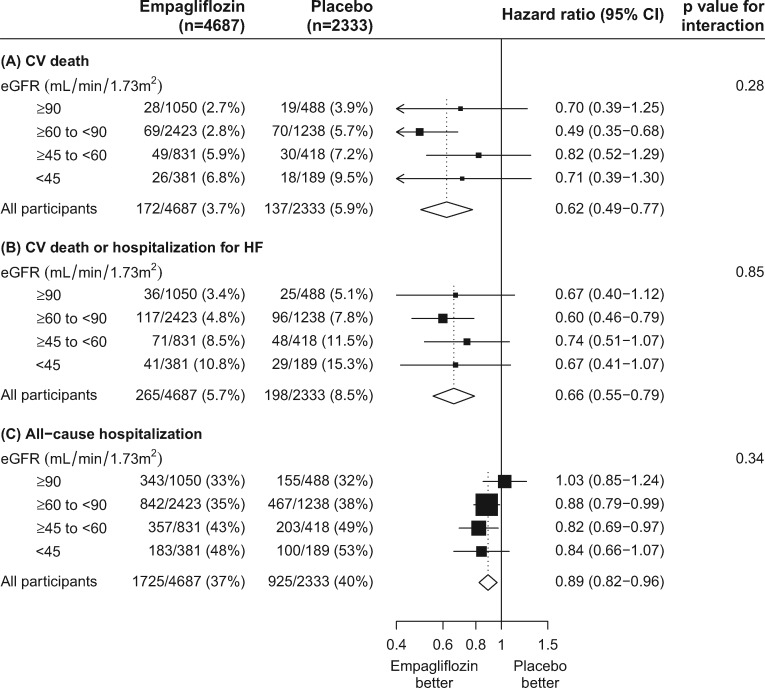
Effect of allocation to empagliflozin versus placebo on (**A**) CV death, (**B**) CV death or hospitalization for HF and (**C**) all-cause hospitalization, by baseline eGFR.

Notably, in subgroup analyses from the EMPA-REG OUTCOME trial, the proportional effects of empagliflozin on CV death and on the composite of CV death or hospitalization for HF were similar irrespective of baseline eGFR (Figure 4A and B) or the level of albuminuria (Supplementary Figure 1) [33, 86].

### Safety and tolerability of SGLT-2 inhibition

In the EMPA-REG OUTCOME trial, empagliflozin was generally well-tolerated during a median follow-up of just over 3 years. The frequency of adverse events that led to discontinuation of study treatment and serious adverse events among participants allocated to empagliflozin was no higher than among those allocated to placebo [32, 33]. Indeed, there was a significant 11% reduction in the risk of hospitalization for any cause among those allocated to empagliflozin compared with placebo [HR 0.89 (95% CI 0.82–0.96); Figure 4C].

**FIGURE 5:**
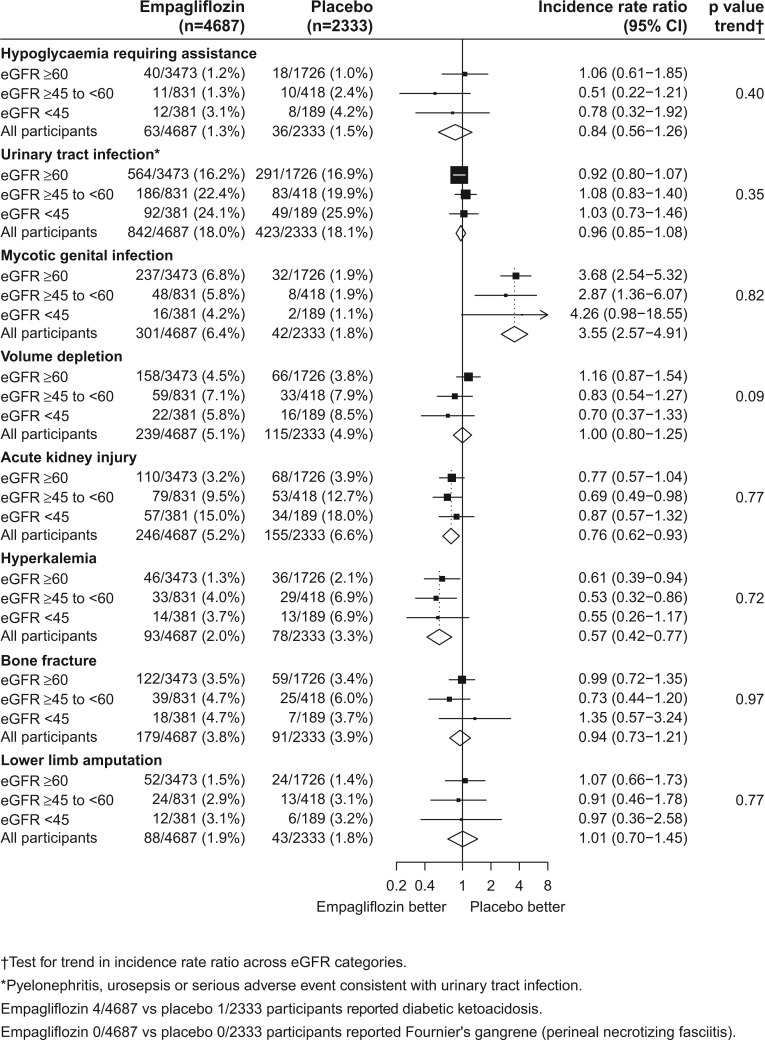
Effect of allocation to empagliflozin versus placebo on adverse events, by baseline eGFR.

Overall in the EMPA-REG OUTCOME trial, there was no significant increase in the frequency of hypoglycaemia requiring assistance among those allocated to empagliflozin as compared with placebo [HR 0.84 (95% CI 0.56–1.26); Figure 5], but there is a potential for increased risk of hypoglycaemia with empagliflozin when used in combination with a sulphonylurea or insulin [87]. Importantly, in studies comprising individuals with normoglycaemia, SGLT-2 inhibitors do not alter fasting plasma glucose levels [55], and so it is not anticipated that SGLT-2 inhibition will increase hypoglycaemia risk in those without diabetes.

All currently marketed SGLT-2 inhibitors carry a warning about diabetic ketoacidosis on their US labels. In the EMPA-REG OUTCOME trial, ketoacidosis was a rare event (see Figure 5 footnote) and so the precise size of the risk of ketoacidosis with SGLT-2 inhibition in different types of people is currently uncertain. Since the most common cause of ketoacidosis is insufficient endogenous insulin availability, the risk of ketoacidosis is expected to be considerably lower in people without diabetes.

The EMPA-REG OUTCOME data showed that, as compared with placebo, empagliflozin increases the frequency of mycotic genital infections by ∼3-fold [HR 3.55 (95% CI 2.57–4.91)] but did not increase urinary tract infections [HR 0.96 (95% CI 0.85–1.08)]. Unlike dual inhibition of the RAS system, the combination of RAS blockade and empagliflozin did not cause serious hyperkalaemia [HR 0.57 (95% CI 0.42–0.77)] or acute kidney injury [HR 0.76 (95% CI 0.62–0.93)], and all these safety assessments appeared similar across the range of baseline eGFRs studied (Figure 5) [13, 33, 86]. Laboratory analyses have also found that blood concentration of calcium and phosphate did not differ in a clinically relevant manner among those allocated empagliflozin versus placebo [87].

The CANVAS/CANVAS-R showed that canagliflozin was also generally well-tolerated [31]. Like empagliflozin, canagliflozin caused an excess of genital mycotic infections, but a possible increased risk of lower-limb amputation and bone fracture was also identified. Neither of these potential hazards were observed in the EMPA-REG OUTCOME trial (Figure 5) or when the EMPA-REG OUTCOME trial was combined with other placebo-controlled empagliflozin trials (including >12 000 participants with T2DM) [87]. Nevertheless, amputations and bone fractures, in addition to ketoacidosis, are being carefully monitored in the ongoing SGLT-2 inhibitor trials.

## CONCLUSIONS

There is a high unmet clinical need to reduce further the risks of kidney disease progression and CV disease in people with CKD irrespective of whether they have diabetes. Results from the EMPA-REG OUTCOME trial and the pooled CANVAS/CANVAS-R programme have raised a strong hypothesis that SGLT-2 inhibition could reduce the risk of kidney disease progression in CKD. Mechanistically, the effects of SGLT-2 inhibition on intraglomerular pressure appear to complement the effects of RAS inhibition (Figure 1) without causing hyperkalaemia or acute kidney injury, so their combination may have the potential to benefit those at risk of hyperfiltration (i.e., those with reduced eGFR and/or albuminuria). Moreover, there is good reason to hypothesize that SGLT-2 inhibition may reduce HF risk, a common condition in CKD. As SGLT-2 inhibition does not appear to require preserved kidney function or overt hyperglycaemia to have important pharmacological effects, another large, prospective, placebo-controlled trial called EMPA-KIDNEY (The Study of Heart and Kidney Protection With Empagliflozin) is now planned in order to test definitively whether SGLT-2 inhibition with empagliflozin can reduce the risk of cardio-renal outcomes in a broad range of people with CKD, including individuals with overt albuminuria and those with low eGFR, irrespective of their level of albuminuria.

## Supplementary Material

Supplementary Data
