# Supplementary material for: The potential for improving cardio-renal outcomes by sodium-glucose co-transporter-2 inhibition in people with chronic kidney disease: a rationale for the EMPA-KIDNEY study
Source: Clin Kidney J. 2018 Oct 25;11(6):749–61. doi: 10.1093/ckj/sfy090 (PMC6275453; doi:10.1093/ckj/sfy090)
Supplement: Supplementary Data [file sfy090_webfigures.pdf]

Webfigure 1: Effect of allocation to empagliflozin vs placebo on (A) cardiovascular death, (B) cardiovascular death or hospitalization for heart failure, and (C) all−cause hospitalization, by baseline urinary albumin:creatinine ratio

	Empagliflozin (n=4687)	Placebo (n=2333)	Hazard ratio (95% CI)	p value for interaction
**(A) Cardiovascular death**				
uACR (mg/g)				0.22
<30	81/2789 (2.9%)	52/1382 (3.8%)	0.77 (0.55−1.09)	
≥30 to ≤300	48/1338 (3.6%)	49/675 (7.3%)	0.49 (0.33−0.73)	
>300	42/509 (8.3%)	36/260 (13.8%)	0.55 (0.35−0.86)	
All participants	172/4687 (3.7%)	137/2333 (5.9%)	0.62 (0.49−0.77)	
**(B) Cardiovascular death or hospitalization for heart failure**				
uACR (mg/g)				0.19
<30	112/2789 (4.0%)	70/1382 (5.1%)	0.79 (0.59−1.07)	
≥30 to ≤300	84/1338 (6.3%)	78/675 (11.6%)	0.54 (0.40−0.74)	
>300	67/509 (13.2%)	50/260 (19.2%)	0.59 (0.41−0.86)	
All participants	265/4687 (5.7%)	198/2333 (8.5%)	0.66 (0.55−0.79)	
**(C) All−cause hospitalization**				
uACR (mg/g)				0.22
<30	923/2789 (33%)	503/1382 (36%)	0.87 (0.78−0.97)	
≥30 to ≤300	543/1338 (41%)	280/675 (41%)	0.96 (0.83−1.10)	
>300	237/509 (47%)	139/260 (53%)	0.77 (0.62−0.94)	
All participants	1725/4687 (37%)	925/2333 (40%)	0.89 (0.82−0.96)	

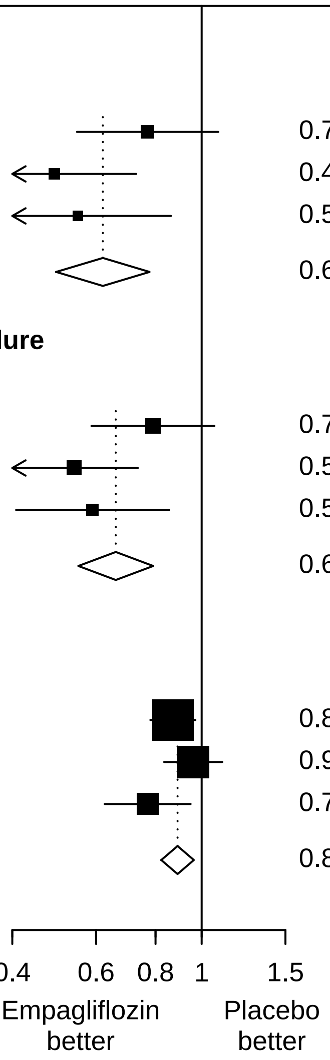

uACR=urinary albumin:creatinine ratio.
